# Antithymocyte globulin administration in patients with profound lymphopenia receiving a PBSC purine analog/busulfan-based conditioning regimen allograft

**DOI:** 10.1038/s41598-020-72415-7

**Published:** 2020-09-21

**Authors:** Maxime Jullien, Thierry Guillaume, Pierre Peterlin, Alice Garnier, Amandine Le Bourgeois, Camille Debord, Beatrice Mahe, Viviane Dubruille, Soraya Wuilleme, Nicolas Blin, Cyrille Touzeau, Thomas Gastinne, Benoit Tessoulin, Yannick Le Bris, Marion Eveillard, Alix Duquesne, Philippe Moreau, Steven Le Gouill, Marie C. Bene, Patrice Chevallier

**Affiliations:** 1 Clinical Hematology, Nantes University Hospital, CHU de Nantes, 1 Place Alexis Ricordeau, 44000 Nantes, France; 2 Hematology Biology, Nantes University Hospital, Nantes, France; 3 EFS de Nantes, Nantes, France

**Keywords:** Immunotherapy, Stem-cell therapies

## Abstract

Graft-versus host disease (GVHD) remains one of the main causes of morbidity and mortality after allogeneic hematopoietic stem cell transplantation (ASCT). Prophylactic T cell depletion via antithymocyte globulin (ATG) during ASCT conditioning is one of the standards of care for GVHD prophylaxis, although the optimal dosing strategy is still unclear. Recent studies have reported that absolute lymphocyte count at the time of ATG administration could predict survivals in ASCT from unrelated donors. Here this issue was examined in 116 patients receiving peripheral blood stem cells (PBSC) ASCT with purine analog/busulfan-based conditioning regimens between 2009 and 2019 in our department. The impact of lymphopenia at the time of ATG administration was evaluated in terms of overall survival, disease-free survival and GVHD-free/relapse-free survival. After a median follow-up of 4 years, no adverse effect of a profound lymphopenia was observed on patients’ outcome. Notably, a reduced dose of ATG in patients with profound lymphopenia did not translate into better survivals. This study indicates that ATG can be administered whatever the recipient’s lymphocyte counts in patients receiving a PBSC purine analog/busulfan-based conditioning regimen ASCT.

## Introduction

Although transplant-related mortality has decreased over the past decades, graft-versus host disease (GVHD) remains one of the main causes of morbidity and mortality after allogeneic hematopoietic stem cell transplantation (ASCT)^1,2^. Antithymocyte globulin (ATG) during conditioning reduces the risk of GVHD by eliminating donor T-cells, but as a consequence the beneficial graft-versus tumor effect may be lessened and the risk for infection increased^3^. The clinical benefit of prophylactic T-cell depletion via ATG has been evaluated in myeloablative (MAC)^4–7^ and reduced-intensity (RIC) conditioning regimen^8–11^, yet with contradictory results. The optimal dosing of ATG thus remains to be determined.

Recent studies have reported that recipient absolute lymphocyte count (ALC) at the time of ATG administration could predict survivals in ASCT from unrelated donors. This suggests that, especially at the cut off of < 0.1 × 10^9^/L, the dose and timing of ATG administration should be adapted^7,12^. To examine this issue, the outcome of ASCT was evaluated in all consecutive patients transplanted for a hematologic malignancy in our department. The impact of lymphopenia at the time of ATG administration was evaluated in terms of overall survival (OS), disease-free survival (DFS) and GVHD-free/relapse-free survival (GRFS) in the particular setting of peripheral blood stem cell (PBSC) purine analog/busulfan-based conditioning regimen ASCT.

## Methods

A retrospective institutional review board-approved analysis was conducted including the 395 consecutive adults transplanted in our department between 01/2009 and 03/2019. The conditioning regimen was purine analogue/busulfan/ATG-based. PBSC only were used as source of graft from matched (MUD) or 9/10 mismatched (mmUD) unrelated donors or siblings. RIC consisted of fludarabine 30 mg/m^2^/day (d) from day 6 to day 2, busulfan 3.4 mg/kg/day from day 4 to day 3 and ATG (Thymoglobulin, Sanofi, Lyon, France) 2.5 mg/Kg/day on day 2 and day 1 (FB2A2) or used clofarabine 30 mg/m CHU de Nantes/day in replacement of fludarabine with 1 or 2 days of ATG (CloB2A1/CloB2A2). Reduced-toxicity (RT) MAC consisted of the same as FB2A2 but with 3 or 4 days of busulfan instead of 2 (FB3A2/FB4A2). All grafts were administered freshly on the day of collection at a time when the patients had already received ATG. GVHD prophylaxis consisted of cyclosporine alone for patients with a sibling donor, while patients grafted with MUD or mmUD received cyclosporine and mycophenolate mofetil.

Patients for whom a blood differential was available at the time of ATG administration were analyzed exhaustively in order to appreciate the impact of lymphopenia on OS, DFS and GRFS. OS was defined as the time from transplantation to death from any cause. DFS was defined as the time from transplantation to disease relapse or death. GRFS was defined as previously published^13^ as the time from transplantation to any event, including grade 3/4 acute GVHD, systemic therapy-requiring chronic GVHD, relapse, or death. Patients alive of lost to follow-up were censored at the last visit.

Statistical analyses were performed using R. The probability of OS, DFS and GRFS was calculated with the Kaplan–Meier estimator, and Log-Rank test was used to compare survivals between groups.

### Ethics approval

This study was approved by the ethical review board of Nantes University Hospital. All procedures were performed in accordance with the ethical standards of the institutional and/or national research committee and with the 1964 Helsinki Declaration and its later amendments or comparable ethical standards. All patients provided informed consent for the retrospective use of their data for research purposes, according to French law.

## Results

Of 395 eligible patients, only 116 were documented with a differential on the day of ATG administration, mostly due to the fact that a differential is not performed daily in our university hospital. Moreover, 9/395 patients had a WBC count between 0.2 and 0.5 × 10^9^/L and were excluded from the analysis due to the technical impossibility to have an exact differential. Yet, 8/395 patients in profound aplasia (WBC < 0.2 × 10^9^/L) were included in the cohort, being considered as having no lymphocytes. Finally, WBC counts between analyzed and excluded patients were similar (respectively 3.75 × 10^9^/L)/L ± 0.45 vs. 3.64 × 10^9^/L ± 0.39, p = 0.72). Patient characteristics are reported on Table 1. RIC was used for 80 (69%) of the patients including 39 FB2A2, 29 CloB2A1 and 12 CloB2A2. RT-MAC was applied for 36 (31%) patients, including 27 FB3A2 and 9 FB4A2. Seventy-six patients had a myeloid disease while 40 had a lymphoid disease. Donor types were siblings (n = 33), MUD (n = 70) or mmUD (n = 13). The median follow-up for surviving patients was 4 years. For the entire cohort, 4 years OS, DFS and GRFS were 56.2% (47–66), 40.9% (32–51) and 34.5% (26–45), respectively (Supplementary Fig. S1). No difference in survivals was observed whether patients initially suffered from a lymphoid or a myeloid disorder (respectively 53.9% vs 56.9%, p = 0.52; 40.8% vs 42.7%, p = 0.34; 27.9% vs 38%, p = 0.14, for OS, DFS and GRFS). No difference either was seen for patients transplanted with sibling vs other donors (64.8% vs 53.2%, p = 0.08; 46.9% vs 40.3%, p = 0.65; 39.5% vs 32.3%, p = 0.40), patients receiving a RIC vs a RT-MAC (52.9% vs 62.3%, p = 0.61; 41.9% vs 43.3%, p = 0.98; 37.8% vs 29%, p = 0.49) or a CloB2 vs a FB2 RIC regimen (48.6% vs 55.5%, p = 0.77; 40.1% vs 42.5%, p = 0.69; 36.8% vs 37.5%, p = 0.48).

**Table 1:** Patient characteristics.

	Total	MAC	RIC
	n = 116	n = 36 (31%)	n = 80 (69%)
Age, years median (range)	59.1 (24.6–73.9)	54.5 (26.1–64.5)	63.7 (24.6–73.9)
Sex, male	71 (61.2%)	22 (61.1%)	49 (61.3%)
**Disease**			
Myeloid neoplasm	76 (65.5%)	22 (61.1%)	54 (67.5%)
AML	49 (42%)	11 (30.6%)	38 (47.5%)
MDS	16 (13.8%)	6 (16.7%)	10 (12.5%)
MPN	11 (9.5%)	5 (13.9%)	6 (7.5%)
Lymphoid neoplasm	40 (34.5%)	14 (38.9%)	26 (32.5%)
ALL	8 (6.9%)	3 (8.3%)	5 (6.3%)
High grade NHL	13 (11.2%)	4 (11.1%)	9 (11.2%)
Low grade NHL	10 (8.6%)	3 (8.3%)	7 (8.8%)
HL	2 (1.7%)	1 (2.8%)	1 (1.3%)
T NHL	7 (6.0%)	3 (8.3%)	4 (5%)
**Disease risk index**			
Low	16 (13.8%)	5 (13.9%)	11 (13.8%)
Int	84 (72.4%)	29 (80.6%)	55 (68.8%)
High	14 (12.1%)	2 (5.6%)	12 (15.0%)
Very high	1 (0.9%)	0 (0%)	1 (1.3%)
**Donors**			
HLA-identical sibling	33 (28.4%)	7 (19.4%)	26 (32.5%)
10/10 MUD	70 (60.3%)	20 (55.6%)	50 (62.5%)
9/10 MUD	13 (11.2%)	9 (25%)	4 (5%)
**Conditionning**			
CloB2A1	29 (25.0%)		29 (36.2%)
CloB2A2	12 (10.3%)		12 (15.0%)
FB2A2	39 (33.6%)		39 (48.8%)
FB3A2	27 (23.3%)	27 (75.0%)	
FB4A2	9 (7.8%)	9 (25.0%)	
**Median ALC/ATG × 10**^**9**^**/L (range)**	0.070 (0–2.3)	0.1 (0.01–1.2)	0.055 (0–2.3)

Unless otherwise specified, data are shown as n(%).

*ALC/ATG* absolute lymphocyte count on the first day of administration of ATG, *AML* acute myeloblastic leukemia, *MDS* myelodysplastic syndrome, *MPN* myeloproliferative neoplasm, *ALL* acute lymphoblastic leukemia, *NHL* non Hodgkin lymphoma, *HL* Hodgkin lymphoma, *MUD* matched unrelated donor.

Median ALC at the beginning of conditioning (day 6) was 0.915 × 10^9^/L (range 0.010–15.780, Supplementary Fig. S2). No difference in terms of survivals was observed when comparing patients under and above this threshold: 4 years OS, DFS and GRFS were respectively of 53.1% vs 59.7% (p = 0.30), 42% vs 42.2% (p = 0.73) and 35.4% vs 33.8% (p = 0.62).

**Figure 1:**
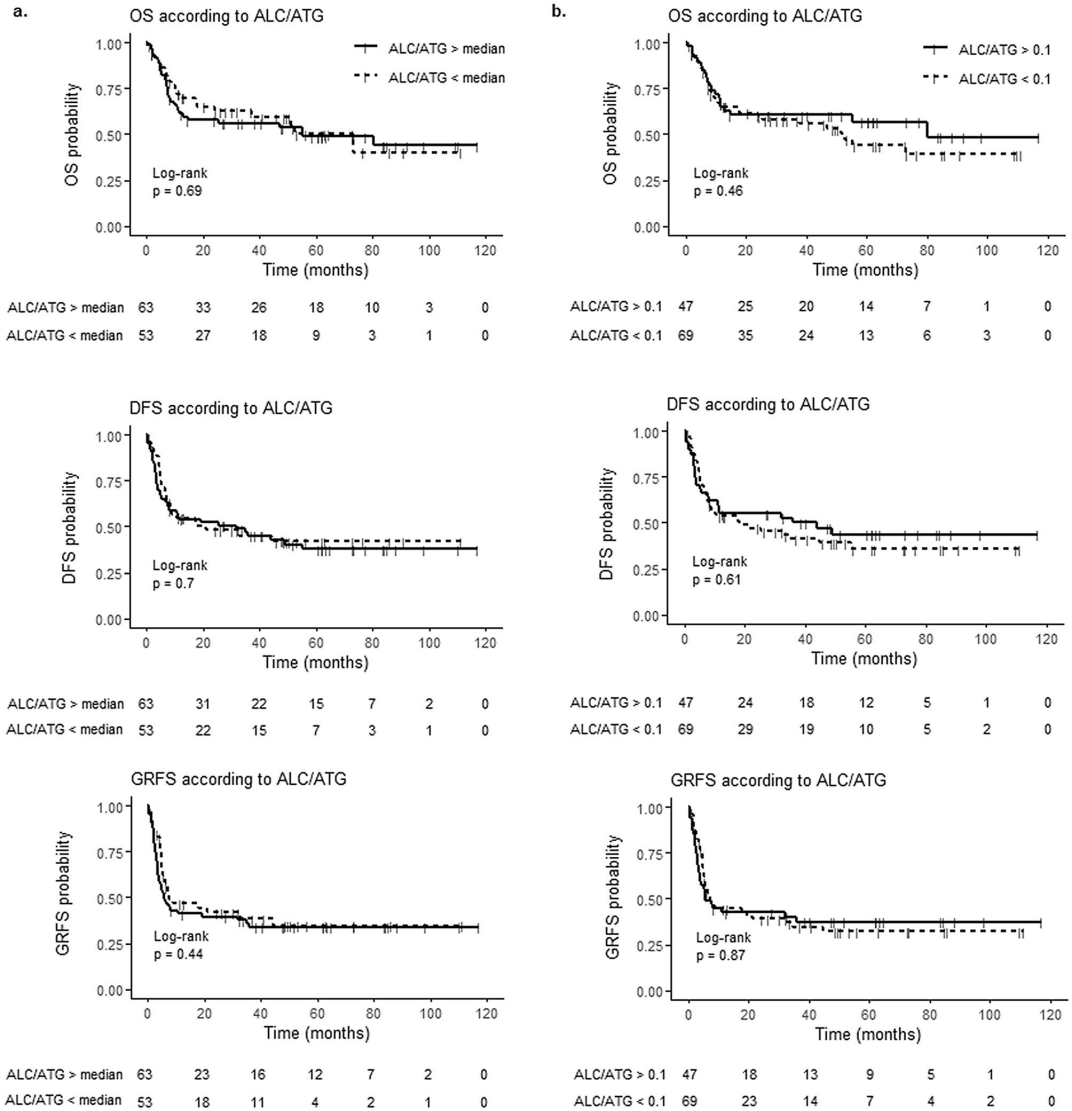
Survivals according to absolute lymphocyte counts at the time of antithymocyte globulin administration (ALC/ATG). (**a**) Cut-off of 0.07 × 10^9^/L lymphocytes. (**b**) Cut-off of 0.1 × 10^9^/L lymphocytes. *OS* overall survival, *DFS* disease free survival, *GRFS* GVHD-free/relapse-free survival.

ROC curve analysis failed to identify a cut-off allowing to predict better survivals according to ALC at the time of ATG administration (ALC/ATG, day 2) (Supplementary Fig. S3). The median ALC/ATG was 0.070 × 10^9^/L (range 0–2.3). Kaplan–Meier curves of OS, DFS and GRFS of patients under and above this threshold are reported on Fig. 1a.

No difference in terms of survivals was observed between both groups: 4 years OS, DFS and GRFS were respectively of 55.1% vs 57% (p = 0.60), 40.2% vs 44.3% (p = 0.74) and 32.4% vs 36% (p = 0.93). The same was true when considering 0.100 × 10^9^/L as ALC/ATG cut-off (Fig. 1b), with 4 years OS, DFS and GRFS respectively of 52.1% vs 61.7% (p = 0.35), 38.3% vs 48.1% (p = 0.53) and 34% vs 38.1% (p = 0.90). Since none of the abovementioned parameters tested displayed any significant impact on survivals in univariate analysis, no multivariate analysis was considered.

**Figure 2:**
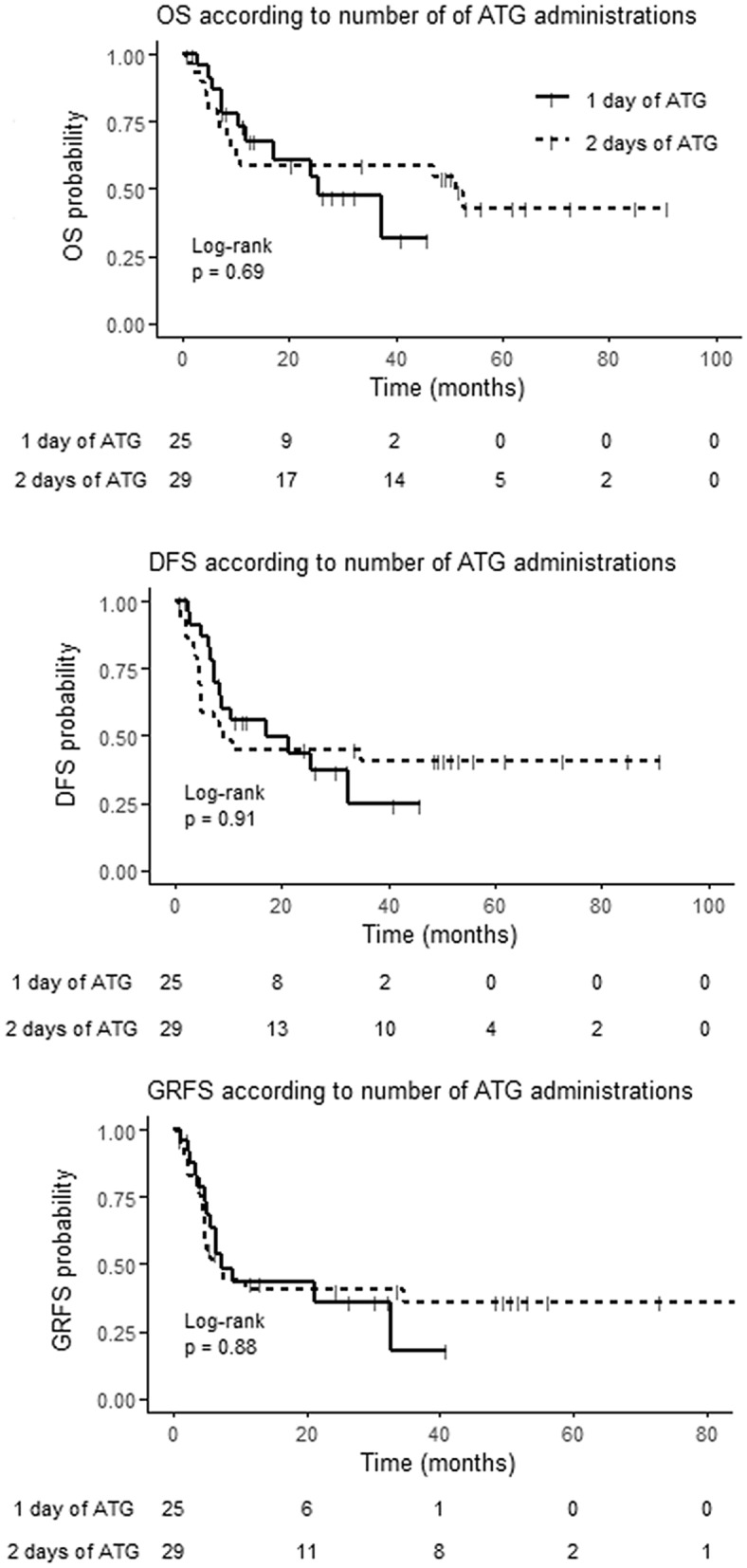
Survivals of patients with reduced-intensity conditioning regimen according to the number of ATG administration. *OS* overall survival, *DFS* disease free survival, *GRFS* GVHD-free/relapse-free survival.

For patients having received a MAC regimen, the median ALC/ATG was 0.100 × 10^9^/L with no difference in survivals between patients under or above this value (Fig. S4). The same was true for RIC using as ALC/ATG cut-offs the median (0.055 × 10^9^/L) or the 0.100 × 10^9^/L threshold (Fig. S5). Interestingly, considering patients with ALC/ATG < 0.100 × 10^9^/L within the RIC setting, survivals were similar between those who received 1 day (n = 25) vs 2 day (n = 29) of ATG (Fig. 2).

Finally, the role of lymphopenia (< 0.1 × 10^9^/L) at the time of ATG administration was studied in both sub-groups of patients receiving ASCT from an unrelated matched (n = 70) or a sibling donor (n = 33), showing that OS (p = 0.50 and p = 0.42), DFS (p = 0.99 and p = 0.20) and GRFS (p = 0.88 and p = 0.24) were not impacted by this criterion. Moreover, even if the number of patients was limited, considering myeloablative allografts with MUD (n = 20), survivals were similar between patients with lymphopenia < 0.1 × 10^9^/L at the time of ATG administration (n = 8) vs others (n = 12) (OS: p = 0.42; DFS p = 0.96; GRFS p = 0.92).

## Discussion

This retrospective cohort analysis aimed at evaluating the impact of absolute lymphopenia at the time of ATG administration as part of purine analog/busulfan conditioning regimens for PBSC ASCT. ALC evaluation at the time of ATG administration is not a routine practice in our department (and probably in many centers), as suggested by the fact that less than 30% of the patients of our cohort were effectively documented with a differential at that time although all obviously had leukocyte counts.

The present study, although retrospective, shows no impact of profound lymphopenia at the time of ATG administration in patients receiving PBSC purine analog/busulfan conditioning regimen ASCT whatever the intensity of the conditioning regimen and the type of donor. The lack of impact of ALC in these patients is emphasized by the fact that a reduced dose of ATG in RIC patients with profound lymphopenia at the time of ATG administration did not translate into better nor worse survivals. However, it must be kept in mind that these results are applicable only in the particular setting of PBSC purine analog/busulfan conditioning regimen ASCT. This may explain the differences observed with other publications.

Indeed, Soiffer et al.^7^ have reported post-hoc analyses suggesting a negative impact of the administration of ATG in patients with ALC < 0.1 × 10^9^/L undergoing myeloablative HLA-matched unrelated ASCT, who had an approximately 25% lower survival rate compared to patients with ALC > 0.1 × 10^9^/L.

Several differences can be noted between the patients of Soiffer’s trial and those reported in this study. Soiffer et al. included recipients of PBSC and bone marrow (BM) stem cells from HLA-matched unrelated donors after a myeloablative conditioning and GVHD prophylaxis consisted of methotrexate and tacrolimus. Here, patients received only PBSC from HLA-identical siblings, MUD or mMUD, and received cyclosporine A + mycophenolate mofetil as GVHD prophylaxis. Moreover, three conditioning regimens were used in Soiffer’s study: cyclophosphamide (Cy)-total body irradiation (TBI), busulfan (Bu)-Cy, and bu/fludarabine (flu). Cy-TBI patients had both low ALC at time of ATG administration and lower OS and DFS compared to patients receiving Bu/flu or Bu-Cy. Additionally, only Cy-TBI patients were impacted by ATG, patients receiving ATG displaying lower OS and DFS. This was not observed for Bu/flu or Bu-Cy patients. This suggests that ATG is maybe only deleterious for patients receiving a Cy-TBI conditioning regimen for ASCT, which may be concordant with our negative results.

Another significant discrepancy between the study by Soiffer et al. and ours is the use of two types of ATG, Fresenius for the former and Thymoglobulin for the latter. This could influence outcomes^14,15^ and thus translate in different impact in lymphopenic allotransplanted patients.

The study by Kennedy et al., which also took into account ALC at the time of ATG administration^12^ reports data close to those of Soiffer et al. in terms of patient characteristics, although these authors used ATG Thymoglobulin and not Fresenius ATG. Of note, a proportion of patients received Cy-TBI as conditioning regimen, which may explain again why our results differ.

Considering our results, we think that other unknown factors rather than recipient lymphopenia remain to be discovered that would justify an optimized individualized dosing of ATG. At the moment, though, the present study indicates that ATG can be administered safely to ASCT recipients receiving PBSC purine analog/busulfan conditioning regimen ASCT whatever the intensity of the conditioning regimen and the ALC at the time of ATG administration.

## Supplementary information

Supplementary Information.
