# Supplementary material for: Antithymocyte globulin administration in patients with profound lymphopenia receiving a PBSC purine analog/busulfan-based conditioning regimen allograft
Source: Sci Rep. 2020 Sep 21;10:15399. doi: 10.1038/s41598-020-72415-7 (PMC7505958; doi:10.1038/s41598-020-72415-7)

# SUPPLEMENTARY MATERIAL

Antithymocyte globulin administration in patients with profound lymphopenia receiving a PBSC purine analog/busulfan-based conditioning regimen allograft.

Authors:

Maxime Jullien[1*], Thierry Guillaume[1], Pierre Peterlin[1], Alice Garnier[1], Amandine Le Bourgeois[1], Camille Debord[2], Beatrice Mahe[1], Viviane Dubruille[1], Soraya Wuilleme[2], Nicolas Blin[1], Cyrille Touzeau[1], Thomas Gastinne[1], Benoit Tessoulin[1], Yannick Le Bris[2], Marion Eveillard[2], Alix Duquesne[3], Philippe Moreau[1], Steven Le Gouill[1], Marie C Bene[2] and Patrice Chevallier[1]

Affiliations:

[1]Clinical Hematology, Nantes University Hospital, Nantes, France; [2]Hematology Biology, Nantes University Hospital, Nantes, France; [3]EFS de Nantes, Nantes, France

Corresponding author:

Dr. Maxime JULLIEN

Hématologie Clinique, CHU de Nantes

1 place Alexis Ricordeau, 44000 Nantes, France

maxime.jullien@chu-nantes.fr

Tel 02.40.08.32.53. / Fax 02.40.08.32.85.

**Figure S1. Survivals in the entire cohort.**

OS: overall survival. DFS: disease free survival. GRFS: GVHD-free/relapse-free survival.

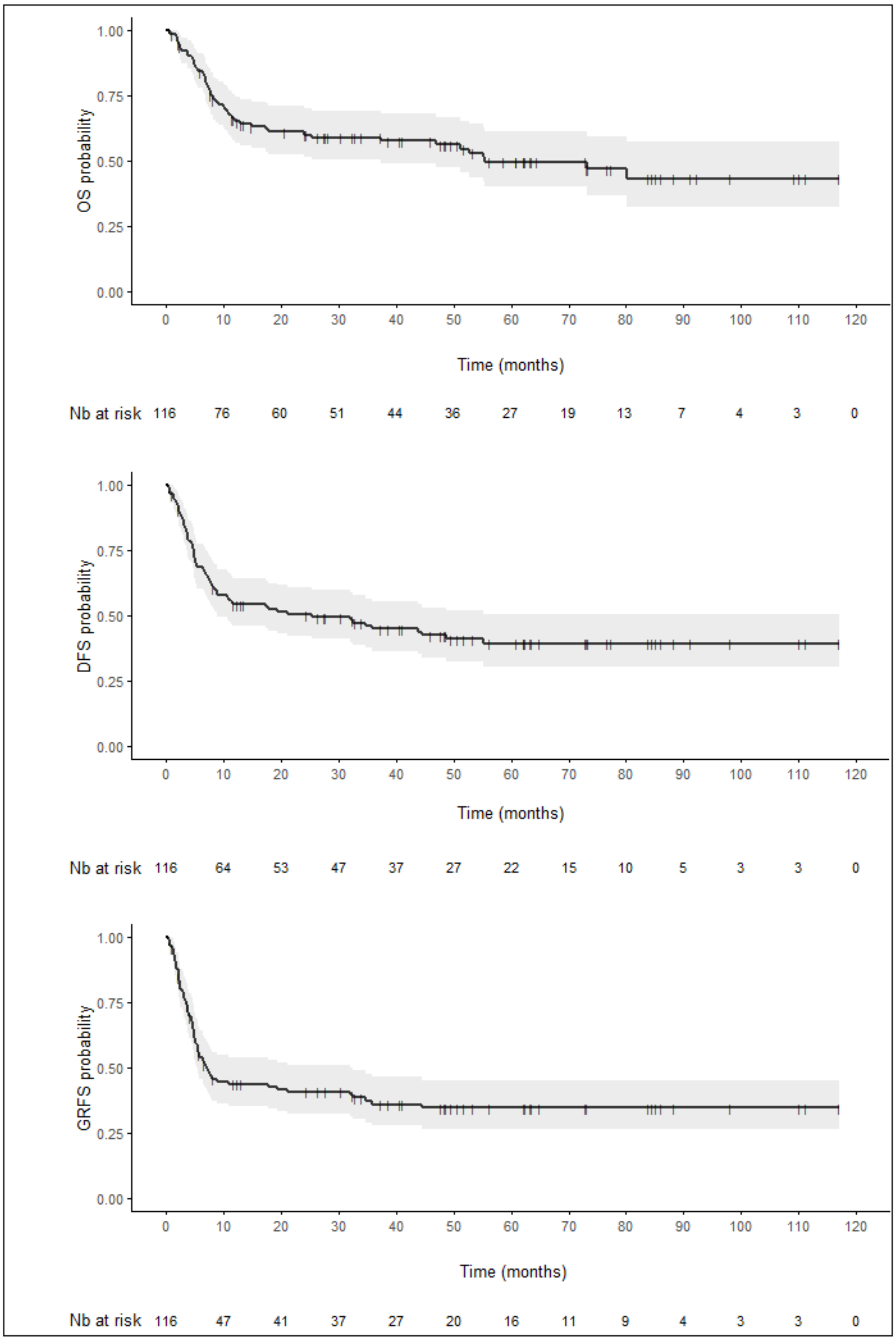

**Figure S2. Absolute lymphocyte count on D-6 (initiation of conditioning chemotherapy) and D-2 (administration of ATG).**

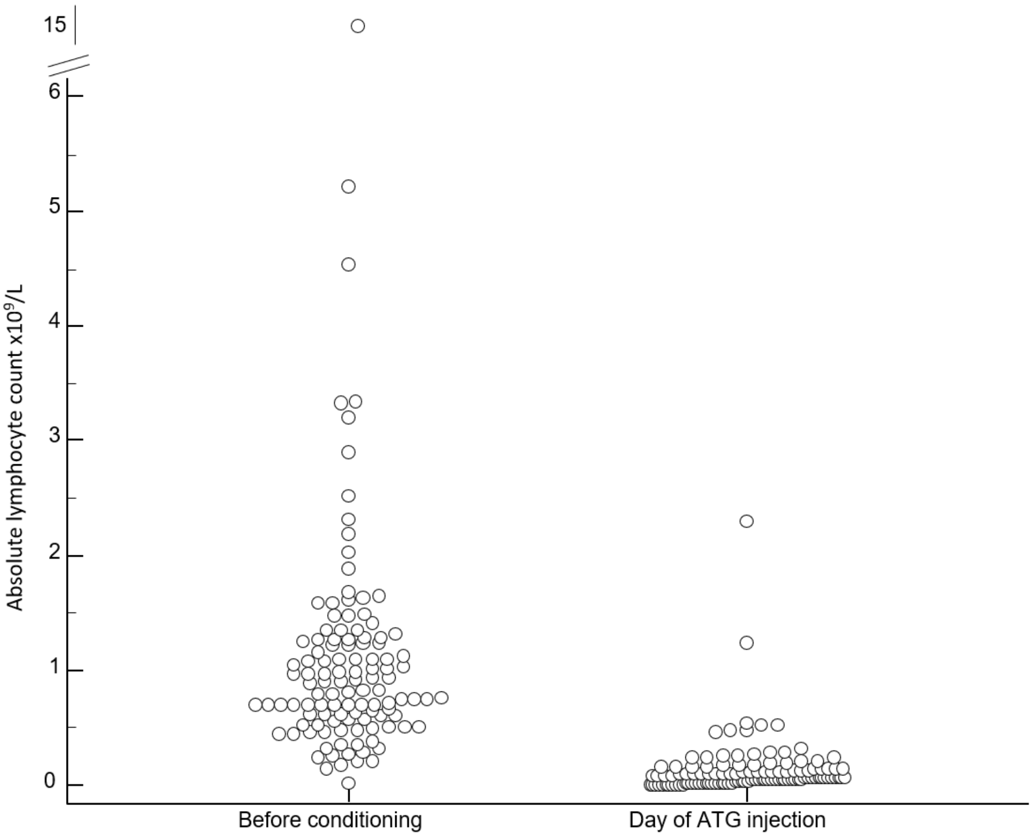

**Figure S3. ROC curves for OS, DFS and GRFS according to according to absolute lymphocyte count at the time of ATG administration (ALC/ATG).**

OS: overall survival. DFS: disease free survival. GRFS: GVHD-free/relapse-free survival. AUC: area under curve.

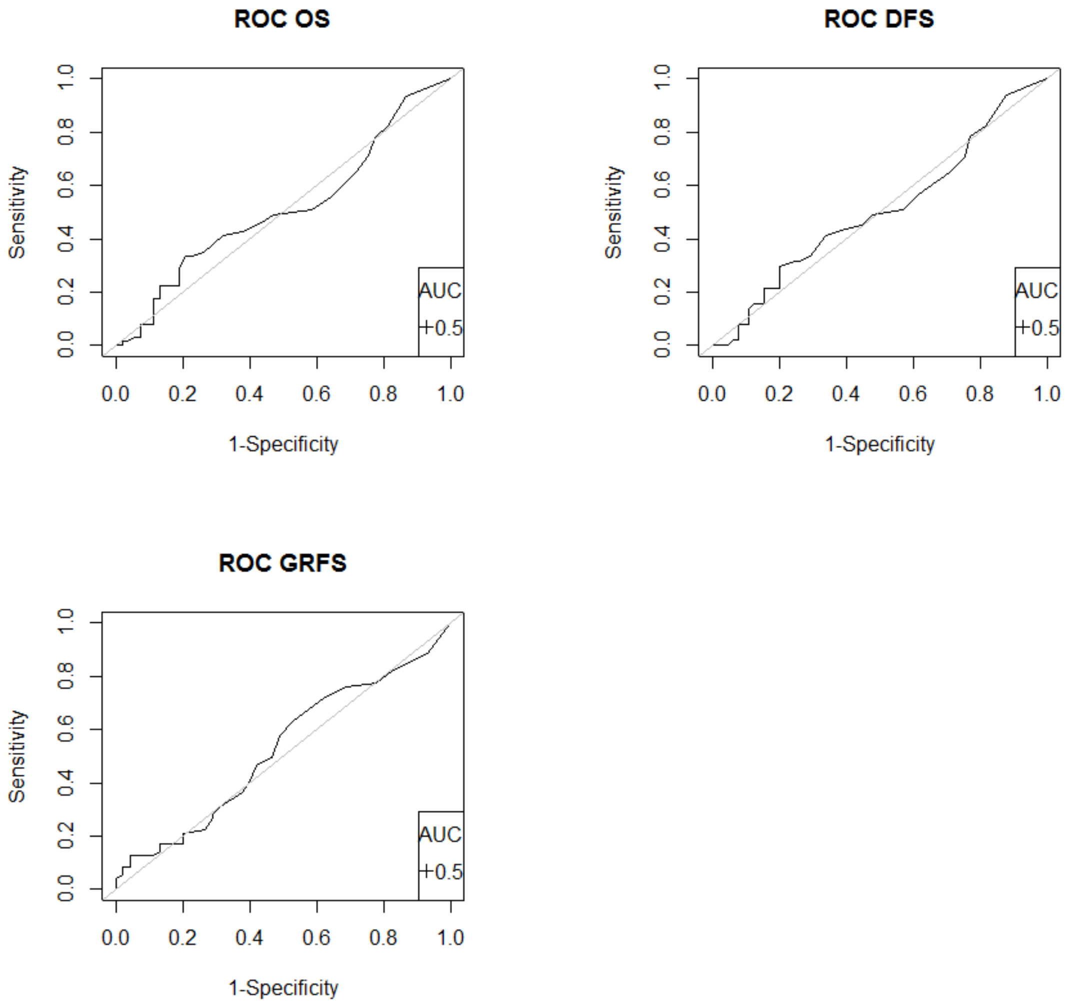

**Figure S4. Survivals of patients with reduced-toxicity myeloablative conditioning regimen according to absolute lymphocyte count at the time of ATG administration (ALC/ATG).** OS: overall survival. DFS: disease free survival. GRFS: GVHD-free/relapse-free survival.

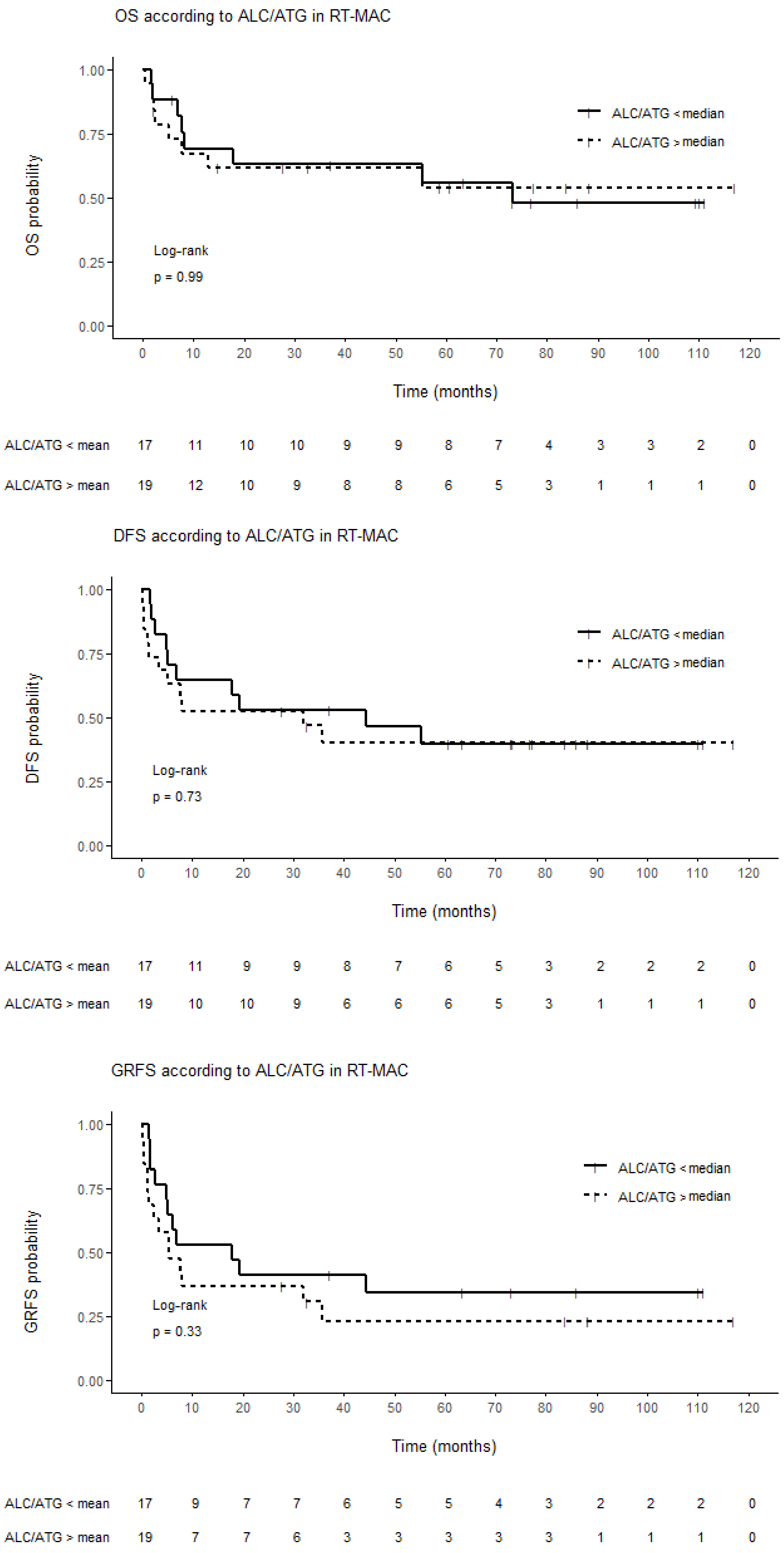

**Figure S5. Survivals of patients with reduced-intensity conditioning regimen according to absolute lymphocyte count at the time of ATG administration (ALC/ATG).**

A: cut-off of 0.055x$10^9$ lymphocytes/L. B: cut-off of 0.1x$10^9$ lymphocytes/L. OS: overall survival. DFS: disease free survival. GRFS: GVHD-free/relapse-free survival.

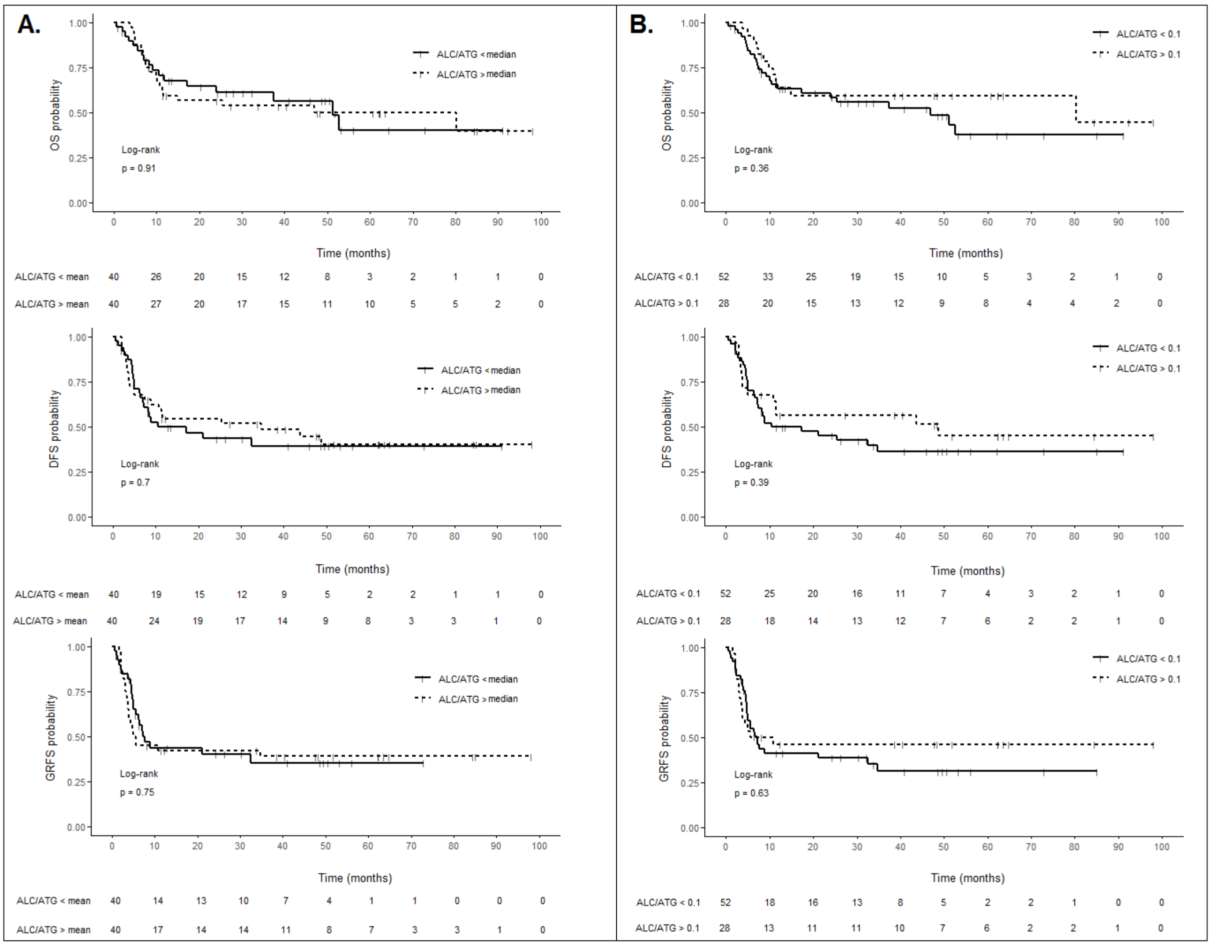

Supplement: Supplementary file 1 — Supplementary Information. [file 41598_2020_72415_MOESM1_ESM.pdf]
